# Supplementary material for: High Levels of ROS Impair Lysosomal Acidity and Autophagy Flux in Glucose-Deprived Fibroblasts by Activating ATM and Erk Pathways
Source: Biomolecules. 2020 May 13;10(5):761. doi: 10.3390/biom10050761 (PMC7277562; doi:10.3390/biom10050761)
Supplement: Supplementary file 1 [file biomolecules-10-00761-s001.pdf]

Supplementary figures

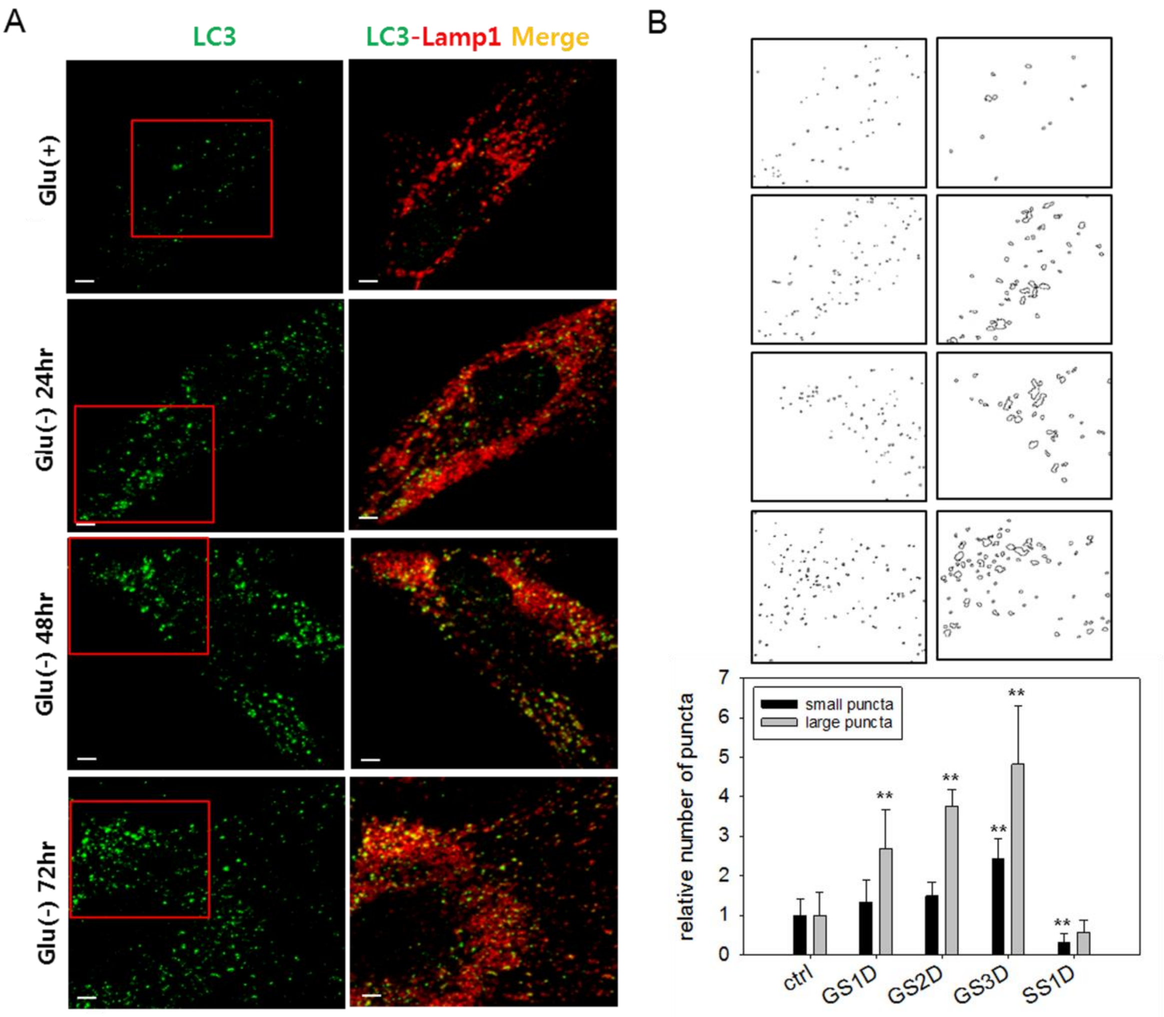

Supplemental Fig 1. Abnormal autophagolysosomes accumulate in glucose-deprived cells. To check co-localization of autophagosomes and lysosomes, cells were visualized after labeling with antibodies against LC3 (green fluorescent) and Lamp1 proteins (red fuorescent) and examined in confocal microscopy. Scale bars in microscopy indicates 5 um. (A). And, the yellow puncta were traced as black and white particle images (produced by thresholding tool of ImageJ (https://imagej.net/Particle_Analysis) and the large and small puncta in more than 40 cells were quantitated (B, upper and lower panels). The numbers of LC3 puncta emerged in cells after 1 day serum deprivation were also plotted (SS 1D). Values are presented as mean ± s.d. * $p < 0.05$, ** $p < 0.01$ by ANOVA.

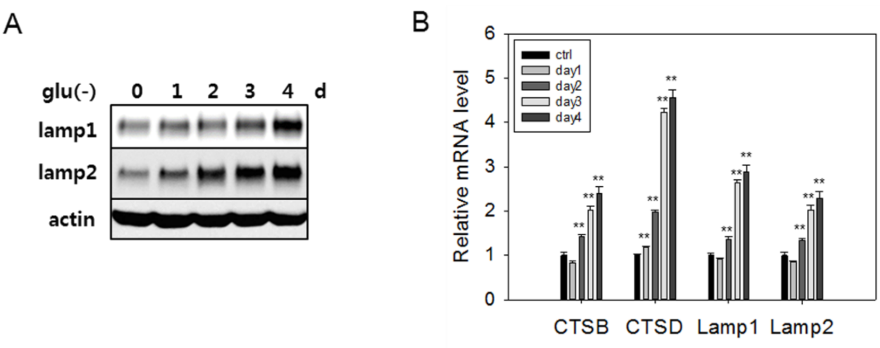

Supplemental Fig 2. Increase in lysosome content and biogenesis in glucose-deprived cells. Cells were incubated in the absence of glucose, and at indicated time points, lysed and either proteins were processed for immunoblot analysis with antibodies against Lamp1 or Lamp2 proteins (A) or RNAs were isolated and processed for quantitative PCR (qPCR) to determine relative levels of mRNA of lysosome genes, cathepsin B (CTSB), cathepsin B (CTSD), Lamp1, and Lamp2. Relative mRNA levels were indicated as the bars in order from day 0 to day 4 (B). Values are presented as mean ± s.d. * $p < 0.05$, ** $p < 0.01$ by ANOVA.

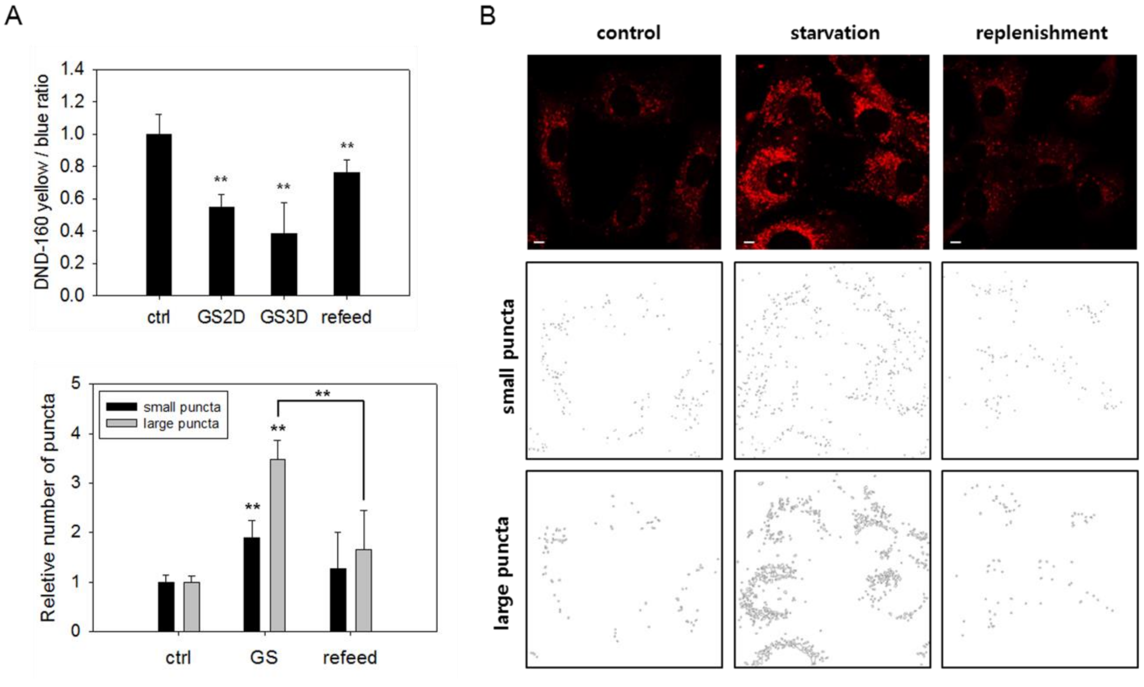

Supplemental Fig 3. Autophagic flux was recovered by glucose replenishment with an increase of lysosomal acidity. Lysosome acidity was measured quantitatively with fluorometry by using Lysosensor DND-160 dye (A). Cells were cultivated in medium deprived of glucose for indicated time. Glucose-deprived cells were supplied with 5.5 mM glucose for the last 24 hours of starvation. Glucose-deprived and glucose-replenished cells were immunostained with LC3 antibody and visualized by confocal microscopy (B). LC3 puncta shown in the photographs were divided into two populations with 1.5-μm diameter criterion and selectively represented below as small and large puncta. Bars in the graph left show the number of LC3 puncta of either small ones (black) or large (grey) Scale bars in photographs indicate 5 μm. Number of puncta was measured in more than 50 cells and plotted by ImageJ software. Values are presented as mean ± s.d. * $p < 0.05$, ** $p < 0.01$ by ANOVA.

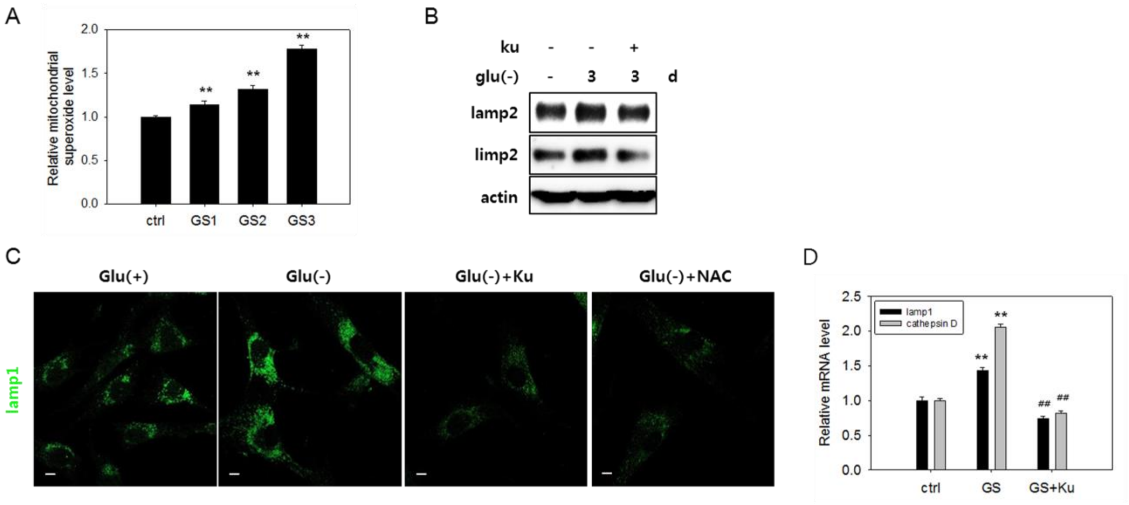

Supplemental Fig 4. ATM inhibition attenuates the increase in lysosome content and lysosome biogenesis in glucose-deprived cells. (A) Cells incubated in glucose-free medium were treated with 0.5 μM KU60019 for three days, and Lamp1 and Limp2 proteins were detected by immunoblotting analysis. (B) Cells treated as in (A) with either 0.5 μM KU60019 or 5 mM NAC were immunostained with Lamp1 antibody and visualized by confocal microscopy. (C) Transcription of two genes encoded by Lamp1 (black bar) or Cathepsin D (grey bar) were determined through qRT-PCR, and presented as bars. # means significance in the difference between KU60019-untreated cells and -treated cells. Values are presented as mean ± s.d. * or # $p < 0.05$, ** or ## $p < 0.01$ by ANOVA.

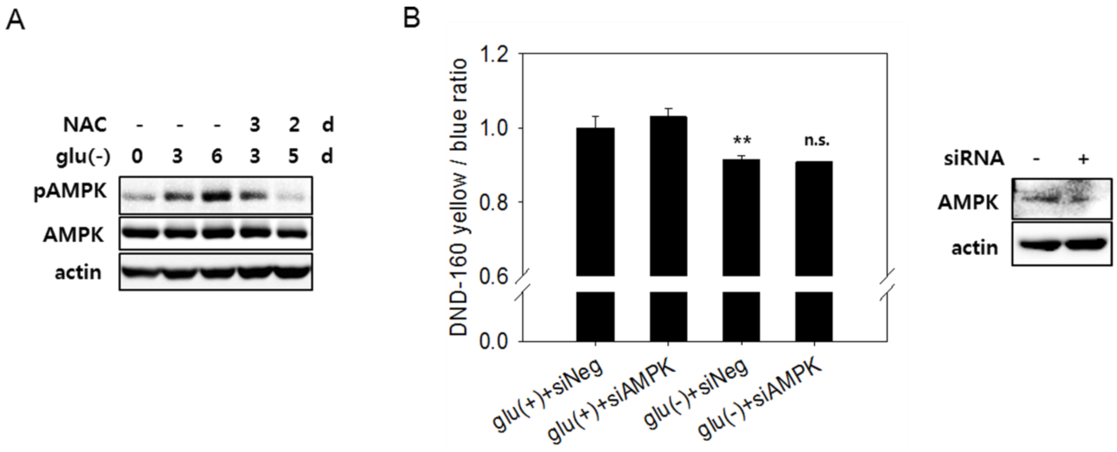

Supplemental Fig 5. AMPK is activated by ROS but irrelevant to lysosomal acidity impairment induced by high level ROS. (A) Cells were cultivated in glucose-free medium for three or six days without or with 5mM NAC for the last three or two days. Activity of AMPK in these cells was determined by immunoblot analysis of AMPK protein phosphorylated Thr172. (B) Cells were cultivated without glucose for two days and transfected with siRNA targeting AMPK mRNA. Cells were further incubated one more day and stained with Lysosensor DND-160 dye to determine acidity of lysosomes. Values are presented as mean ± s.d. * $p < 0.05$, ** $p < 0.01$ by ANOVA.
